# Implementation of Facility Management for Port Infrastructure through the Use of UAVs, Photogrammetry and BIM

**DOI:** 10.3390/s21196686

**Published:** 2021-10-08

**Authors:** Constanza Jofré-Briceño, Felipe Muñoz-La Rivera, Edison Atencio, Rodrigo F. Herrera

**Affiliations:** 1 School of Civil Engineering, Pontificia Universidad Católica de Valparaíso, Valparaíso 2340000, Chile; constanza.jofre.b@mail.pucv.cl (C.J.-B.); edison.atencio@pucv.cl (E.A.); rodrigo.herrera@pucv.cl (R.F.H.); 2 School of Civil Engineering, Universitat Politecnica de Catalunya, 08034 Barcelona, Spain; 3 International Center for Numerical Methods in Engineering (CIMNE), 08034 Barcelona, Spain

**Keywords:** port infrastructures, facility management, building information modelling (BIM), unmanned aerial vehicles (UAVS), photogrammetry, visual programming

## Abstract

The maintenance of port infrastructures presents difficulties due to their location: an aggressive environment or the variability of the waves can cause progressive deterioration. Maritime conditions make inspections difficult and, added to the lack of use of efficient tools for the management of assets, planning maintenance, important to ensure operability throughout the life cycle of port infrastructures, is generally not a priority. In view of these challenges, this research proposes a methodology for the creation of a port infrastructure asset management tool, generated based on the Design Science Research Method (DSRM), in line with Building Information Modeling (BIM) and digitization trends in the infrastructure sector. The proposal provides workflows and recommendations for the survey of port infrastructures from UAVs, the reconstruction of digital models by photogrammetry (due to scarce technical documentation), and the reconstruction of BIM models. Along with this, the bidirectional linking of traditional asset management spreadsheets with BIM models is proposed, by visual programming, allowing easy visualization of the status and maintenance requirements. This methodology was applied to a port infrastructure, where the methodology demonstrated the correct functionality of the asset management tool, which allows a constant up-dating of information regarding the structural state of the elements and the necessary maintenance activities.

## 1. Introduction

Port infrastructures (PI) are large-scale facilities with certain particularities that differentiate them from others designed in less aggressive environments [1]. They exist in a highly aggressive environment, and materiality, the presence of salt water, and the impact of coastal dynamics affect infrastructures, accelerating the progressive deterioration of the elements that compose them. Additionally, environmental conditions make it difficult to inspect and maintain and/or repair this type of infrastructure [2]. The importance of maintaining port infrastructure lies in its strategic role in the development of economic activity or connectivity, which could change over time and convert to recreational activities, although the operability and safety of the infrastructure must be guaranteed throughout its life cycle [3].

Currently, maintenance activities for this type of infrastructure are affected by the difficulty of access, the lack of existing documentation (since many of these structures are old), and the materiality of the elements that compose it (usually reinforced concrete), problems compounded by the marine environment [4], increasing the likelihood of developing anomalies that may cause progressive damage to the infrastructure [2,5]. However, traditional methods for the maintenance of port infrastructure generally follow a corrective maintenance approach with the clear identification of a deterioration, which is also often inefficient due to poor information management [6].

Currently, the new methodologies that have been incorporated into the AECO (Architecture, Engineering, Construction, and Operations) industry encourage and allow the development of preventive infrastructure maintenance [7]. Among the new management trends, facility management (FM) is a discipline that seeks to manage the proper functioning of buildings and/or infrastructures through the integration of people, space, processes, and technologies [8], which translates into optimal service management, operational management, and maintenance management, among others [9,10]. 

The use of building information modelling (BIM) allows the visualization of a project and the integration of all agents and assets for the successful development of the facilities, addressing their entire life cycle. The use of parametric 3D models allows for identification of the typologies of elements that make up the infrastructures, and having full control of the infrastructures as far as the information of materiality, cost planning, time, and environmental considerations that allow greater control and efficiency in the design, construction, and operation processes of the products of this industry [7]. 

However, the use of technologies such as unmanned aerial vehicles (UAVs) allows for the surveying of environments, reaching areas with difficult access, and their 3D digital reconstruction by photogrammetry [11]. From the photographs captured with UAVs and the processing of images in software based on structure from motion (SfM) and multiview stereo (MVS) techniques, it is possible to reconstruct real scenarios and three-dimensional models.

Faced with the difficulties and problems presented by port infrastructures and the new methodologies and technologies that have become widespread in the industry, this investigation proposes a workflow for the integration of the FM activities into harbour infrastructures. We propose to survey existing infrastructure by using UAVs, with reconstruction by image processing based on computer vision algorithms SfM-MVS to generate a point cloud which, together with existing map documentation (generally scarce in old infrastructures), allows for rebuilding parametric 3D models of port infrastructure in BIM modelling software, and facilitates visual and interactive management. The proposed flow also allows for the bidirectional linkage of the information flows, generating an automatic asset management tool based on 3D models and user-friendly maintenance sheets that can be used in the field for port asset maintenance inspection.

The methodology developed was applied to a case study of a port penetration infrastructure, in which the feasibility of implementation was evaluated, the flow of bidirectionality between the tools created was studied, and the main anomalies of infrastructure elements according to typology and materiality were studied to develop an effective asset management tool for the infrastructure in question. 

## 2. Research Methodology

**Figure 1:**
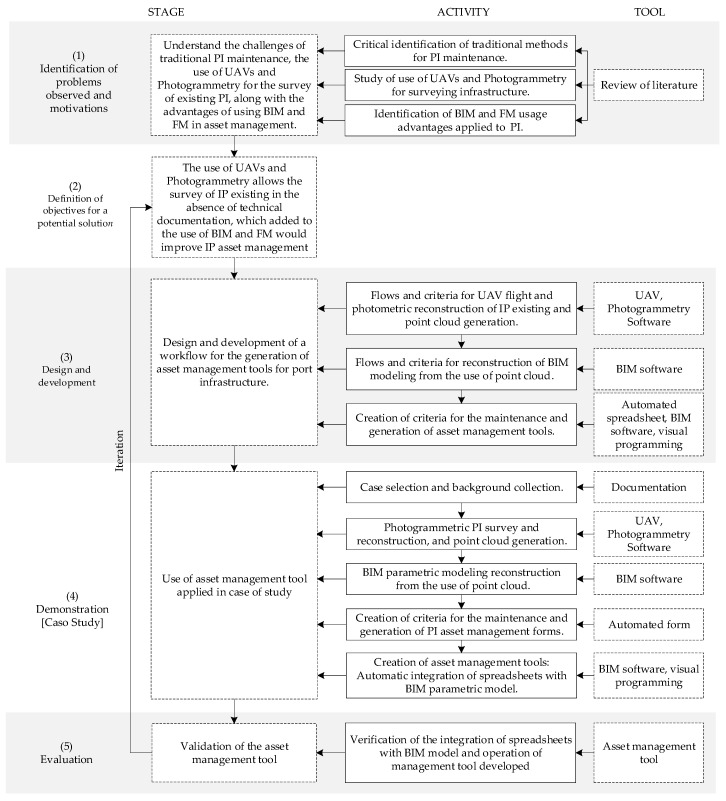
Methodology of research.

This research studies and proposes the use of technologies and new methodologies to optimize the asset management of existing port infrastructure. Figure 1 details the activities needed to carry out this research, together with the tools and methodologies used in its development. The methodology of research in Design Sciences (Design Science Research Methodology—DSRM) has been used to represent the research process practically, organized in 5 stages: (1) identification of observed problems; (2) definition of objectives for a potential solution; (3) design and development; (4) demonstration; (5) evaluation [12].

In the first stage, a literature review was carried out based on Web of Science and Scopus repositories, along with the revision of manuals, guides, and technical reports, to identify deficiencies and particularities of the traditional methods of maintenance of port infrastructures and to define the challenges that exist in the management of their maintenance. Along with this literature review, the potentialities of emerging technologies and methodologies that have been incorporated into the AECO industry were studied: use of UAVs for the collection of information, photogrammetry for the reconstruction of existing infrastructure, and the use of BIM and FM system for information management applied to coastal infrastructure. Based on the background gathered, in the second stage, the objective of a potential solution to the identified problems has been defined: the use of UAVs and photogrammetry allow for the lifting of existing infrastructure, in the absence of documentation regarding its structuring, which, combined with the use of BIM and FM tools, would improve asset management, filling in the gaps in the traditional port infrastructure maintenance methods.

In the third stage, the design and development of a set of tools for maintenance management based on the use of UAVs, photogrammetry, visual programming tools, and BIM software, for the reconstruction of a digital parametric 3D model of port infrastructure interconnected with management worksheets, identifying control elements, and defining conservation activities, make it possible to develop an efficient and practical asset management tool for users.

In the fourth stage, the tools designed and developed in the previous stage are applied to a case study of a port infrastructure that has had little maintenance and has a lack of information about its state and structure. Here, background and documentation of the infrastructure are collected, which provide information of interest for the reconstruction of the BIM parametric model. The parametric BIM model is made from a point cloud obtained from photogrammetry with UAV, thanks to the use of structural from motion (SfM)-multiview stereo (MVS) algorithms of the image processing software. However, maintenance criteria are created associated with the types of the element identified from the parametric BIM model created (in Autodesk^®^ Revit), existing documentation, and field inspections, for which automated forms are developed (in MS Excel) that allow the structural evaluation of the elements and their corresponding maintenance activities. These spreadsheets are integrated into the parametric BIM model using visual programming tools (Dynamo), which allow for the automatic flow of information between both platforms, thus generating an asset management tool that addresses the need to carry out repair activities in a systematic manner to prevent significant deterioration of facilities and that, in turn, ensures an improvement in the management of maintenance processes.

Finally, in the fifth stage, the tools developed are validated, confirming the correct integration of the parametric BIM model with the automated spreadsheets and their usability, thus validating the functioning of the asset management tool.

## 3. Literature Review

Port infrastructures are maritime engineering structures intended for cargo and/or passenger transfer operations due to their function of connectivity between the maritime and land sectors. Within these sectors, there are different typologies according to their orientation or impact on coastal dynamics [2]. These structures are constantly exposed to a marine environment; therefore, they have specific properties that differentiate them from infrastructures designed in less aggressive environments [1,13]. Major damage to port infrastructure results in costs for the repair of the structure itself and economic losses associated with loss of its economic activity due to inoperability while the repair work is being carried out [14]. Early detection of the potential structural pathologies of the infrastructure elements and early performance of the maintenance activities avoids a progressive deterioration, ensures its functionality, and ensures users’ safety. Early maintenance avoids incurring the high costs incurred in financing complex repair work in the event of collapse or large-scale breakage [1,2], reducing the risk of collapse in more complex situations, such as large seismic events and/or maritime phenomena such as tsunamis [15].

### 3.1. Problems and Challenges for Port Infrastructure Maintenance

The complexity of the marine environment has prompted expert entities to develop technical guides that establish a series of standards for port infrastructures [2,5]. These strategies are associated with the material of the elements (usually concrete and steel), the use of anticorrosion elements and the technical characteristics of the materials used in the design, construction practices, and maintenance considerations, among others.

Structural concrete, commonly used for PI due to its strength and versatility, is vulnerable to high concentrations of chlorides and sulfates in coastal waters [16,17], leading to the corrosion of armour and loss of concrete strength [18,19], and, as a result, cracks and breakdowns of concrete occur and, in more extreme cases, could lead to the collapse of the structure [5,11]. The level of corrosion of the armour is relative to the aggressiveness of the environment in the maritime exposure zones. Depending on the different levels of aggressiveness, different pathologies will develop that affect to different degrees the elements that make up the infrastructure [20,21].

In this sense, the importance of detecting PI pathologies promptly lies in the prevention of major deterioration that could affect the functioning of PI. However, PI inspection and monitoring studies are not simple. The presence of water hinders access to inspect the infrastructure in detail throughout its length and depth, which, added to the aggressive environment and high humidity, requires specialized equipment and tools to be used in inspection or repair activities to enable them to be carried out smoothly [22].

### 3.2. Traditional Methods of Port Infrastructure Maintenance

Traditionally, to carry out a maintenance strategy for port infrastructure, there are four main phases: (I) analysis of existing documentation; (II) inventory; (III) overall evaluation; (IV) record of actions [1]. Phase I consists of the analysis of all existing information (plans, photographic records, repair history, among others). In phase II, through visual field inspection, all elements are identified, and the geometrical and technical characteristics of each of the elements are recorded in inventory sheets. In phase III, information is obtained on the status of the PI assets, and the anomalies of each of them are recorded to subsequently define the repair techniques and the urgency of intervention required. Finally, in phase IV, the repair actions carried out for the updating of the state of the infrastructure are recorded [2,18].

These traditional methods are based on the collection and documentation of information on assets on the ground; however, the large size of port infrastructures makes these processes time-consuming [2]. The information collected is recorded in cadastres or inventory sheets, which generally include graphic references or indications associated with existing plans to reference the location of the elements and to facilitate the location of the damage [23]. In many cases, this information is digitized, which facilitates access to existing documentation; however, in this process, digitalization of the cards induces errors [24]. This delay accounts for the shortcomings of traditional methods based on physical or digital records that merely have an orderly record, missing the potential of new methodologies and technologies to manage and visualize the information and documentation collected [25]. In addition to visual inspection, on-site or laboratory tests are often carried out to define the structural state of the infrastructural elements. Based on the results of these tests, it is possible to avoid errors of arbitrary assessment that could be generated in maintenance procedures based solely on visual inspection because, for example, pathologies such as corrosion of the internal reinforcement system of the concrete are not visible [15]. 

In particular, carrying out inspections, testing, and, in general, gathering information to develop conservation strategies becomes complex with regard to infrastructure at sea. Sometimes, underwater inspections are required, which are carried out by professional divers using specialized tools and equipment to measure damage and anomalies in submerged elements [1]. In addition, if the infrastructures are categorized under patrimonial safeguards, there is an increase in restrictions and care with the elements that make up the structure, which, added to the scarce existing documentation, which is generally obsolete, hinders actions to establish maintenance plans under traditional criteria. The damage is therefore not detected in time [26]. Added to these problems due to the nature of PI, there are economic and human resources, recruitment, and use of technologies, which make it difficult to develop efficient conservation management [1].

### 3.3. New Technologies for the AECO Industry

The new methodologies and technologies that have been incorporated into the AECO industry have made it possible to optimize workflows through tools that allow for collaboration and the integration of information, thus evolving the traditional methods [27].

The BIM allows for integration and collaboration between stakeholders in a project through parametric models at different levels of detail and can be used throughout the life cycle of a project [9]. In addition to three-dimensional visualization, with potential access to information, the BIM methodology facilitates the updating of the model, either of parameters or plans of the different specialties without loss of information [28]. In this way, once the model has been developed, it is possible to modify parameters as well as add and/or remove elements as required to have a completely updated and reliable model that can be accessed by all project agents, rendering the traditional systems obsolete in which the projects are worked from the design until the O&M phase, including the manual registry of records of cadastres or conservation, the fragmented documentation of each element of the infrastructure, and the recording and updating of maintenance activities [7,29,30].

In line with the new methodologies, facility management allows for the management of the correct functioning of buildings or infrastructures. The International Association of Facility Management defines “FM” as a discipline that encompasses various areas to ensure and manage the operation of buildings and/or infrastructures and their associated services through the integration of people, spaces, processes, and technologies specific to such buildings or infrastructures [31]. In this sense, correct and reliable information management facilitates decision-making in the operation and maintenance (O&M) phase [32]; however, traditional FM activities methods are performed based on planes or fragmented or dispersed information, which requires more time to be spent collecting information due to the lack of integration of information [33]. Thus, the integration and use of BIM in FM is becoming increasingly common. Currently, it has been used for the maintenance of building assets or large infrastructures, facilitating access to information from the digitisation of the BIM model, which improves the levels of efficiency and productivity of the sector by optimising information flows through existing interoperability formats [8], optimising and significantly reducing costs in the O&M phase [34] so that such integration becomes a potential for efficient asset management [35].

Moreover, new technologies have emerged in the AECO industry to solve the problems of access, information gathering, and digital reconstruction of existing infrastructures [26,36]. Among these technologies, terrestrial laser scanners (TLSs) are mass point acquisition tools which are very useful for the reconstruction of digital models from point clouds [37,38,39]. While this tool allows for massive, fast, and accurate data capture, generating a cloud of high-resolution points, these types of tools are high-cost equipment and require highly trained professionals for their operation, therefore their massive use is restrictive. On the other hand, unmanned aerial vehicles (UAVs) are remotely operated aerial vehicles that have become a common method for mapping and digital reconstruction of infrastructures that, due to their economy and practicality, are preferred over the use of TLSs [40]. Unlike the traditional visual inspection of maintenance plans, the use of UAVs allows for inspections in much shorter times, in turn allowing for more frequent inspections, increasing the recurrence of the structural status updates of the elements of the inspected infrastructure, and thus providing the information for the digital model [11].

The reconstruction of digital models from photogrammetry is based on the SfM technique, which allows for the generation of digital reconstructions from photographs. SfM generates a point cloud, which is improved by densifying its resolution through MV. This technique depends on the quantity and quality of the captured images and the conditions of the environment at the time of capture. Light conditions, projected shadows of objects, and areas of homogeneous surfaces can alter the 3D reconstruction, from which it is possible to generate digital models of the infrastructure [41].

## 4. Methodology Developed

**Figure 2:**
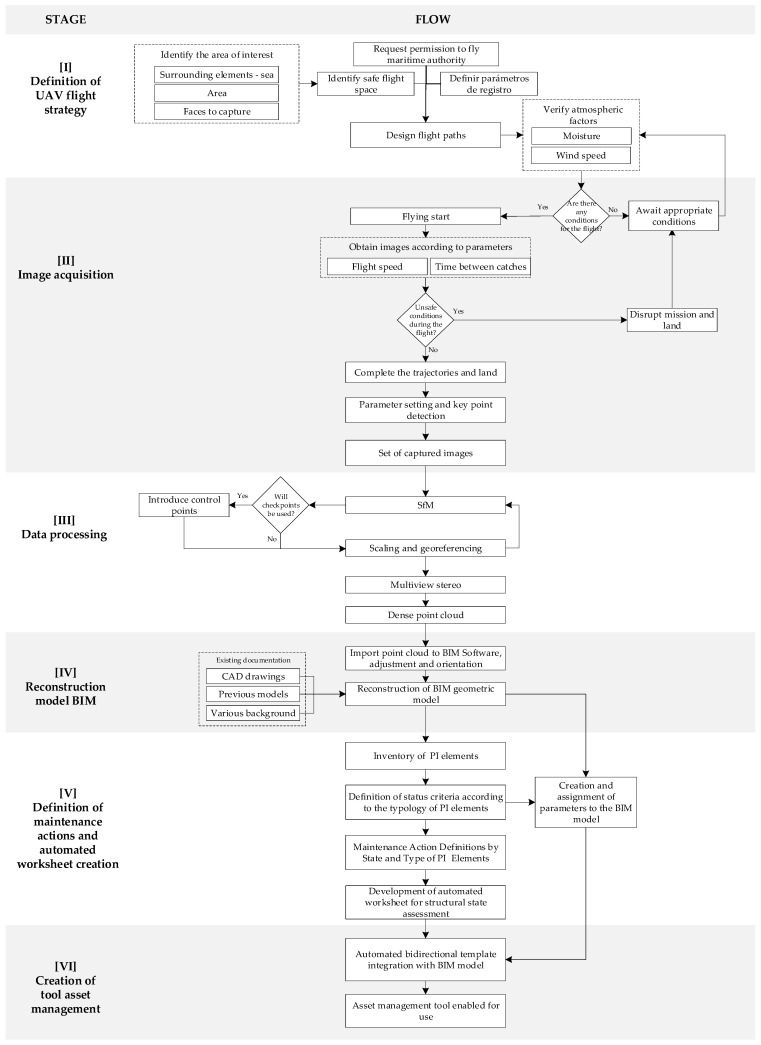
Flow for the creation of an asset management tool.

Figure 2 shows the proposed methodology for generating an asset management tool for port infrastructure, consisting of six stages: (I) UAV flight strategy definition; (II) image acquisition; (III) data processing; (IV) BIM model reconstruction; (V) definition of maintenance actions and automated template creation; (VI) creation of asset management tools. This section shows the conceptual aspects of the proposal. The tools used and integrated into the workflow and the codes developed are shown in detail in the application case section.

### 4.1. Definition of Flight Strategy

UAV flight strategy for PI uplift must be defined. Because PIs are considered strategic infrastructures (private or public, as appropriate), it is necessary to request the corresponding permits to conduct the flight of the UAV, according to the general flight rules of these devices, and according to maritime space registry records established in each country, as appropriate (usually coastal infrastructure is under naval security). In Chile, UAV flight is regulated by the Directorate General of Civil Aeronautics (DGAC), which currently has two regulations that establish certain requirements and limitations for aerial activity: DAN 151: Remotely Piloted Aircraft Operations (RPAS), used in matters of public interest, which take place over populated areas; DAN 91: Rules of the Air, which, unlike the former, applies to non-populated areas. 

It is worth mentioning that those UAVs whose weight exceeds 750 g must comply with DAN 151, which indicates the maximum weight, establishes who can pilot them, the conditions under which they must be operated, the obligation to register with the DGAC, and the documents required for their use. 

On the other hand, there are two cases of UAVs weighing less than 750 g. When used in populated places at an altitude of fewer than 50 m, they are not subject to DAN 151 in terms of registration, credentials, and authorization; however, the operator is responsible for any damage to third parties. However, those used in non-populated areas must only have the corresponding authorization from the DGAC, in accordance with the provisions of DAN 91 [42,43]

Then, the area of interest must be identified. This methodology has been developed for the port infrastructure of piers of penetration, that is, when the PI is projected from the coastal edge towards the sea (being able to adapt to other topologies according to the requirements of the user). In these cases, it is relevant:

- To identify all surfaces and faces of interest that are necessary to rebuild; therefore, it is recommended that the set of photographs cover the entire surface of the infrastructure and its perimeter area, covering the greatest possible visibility of the elements undercover;
- For a correct reconstruction, it is necessary to correctly define the registry parameters that the UAV will use. In practical terms, it is important to consider an overlap between photographs by over 75%;
- To consider the surrounding or PI-specific elements that might interfere with the flight and the difficulties of the near-sea flight (especially when capturing items that are under or near the water line at the time the logs will be taken);
- Atmospheric factors should be considered in the planning of the day of the flight (low humidity and wind speed).

**Figure 3:**
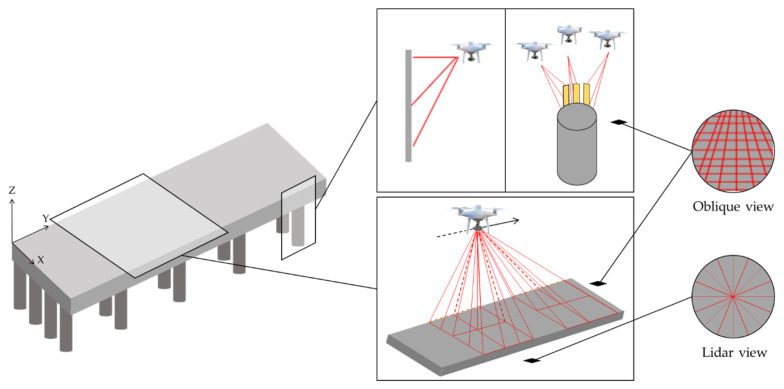
UAV flight path and photogrammetry used.

Under these considerations and given the characteristics of the penetration springs, as shown schematically in Figure 3, it is recommended that the flight path of the UAV covers the surface of the infrastructure with vertical photogrammetry, that is, the axis of the camera positioned vertically with a flight parallel to the surface, which, for a better reconstruction, can be complemented with oblique photogrammetry (especially if there are many elements of complementary infrastructure on the dock). However, oblique photogrammetry is recommended for the entire perimeter of the infrastructure, trying to secure three different directions, to achieve greater coverage of the elements. 

It is important to consider that the homogeneity of the sea surface and the recurrence of similar elements in the PI can generate problems in later photogrammetric reconstruction; therefore, it is recommended to define milestones in the PI that allow for the identification of different points to make potential corrections in the reconstruction.

### 4.2. Data Acquisition

The acquisition of images in the field with the UAV must be done. Atmospheric factors must first be verified. Flights should not be made on rainy days with high humidity and/or strong winds, as these conditions affect the functionality of the equipment or destabilize its position, preventing the acquisition of photographs with the required quality (in capture and positioning). A good photogrammetric reconstruction requires a scenario with few shadows; therefore, schedules must be found where these conditions exist. A mid-day capture is ideal, as the position of the sun is practically perpendicular to the surface, and, therefore, the projected shadow of the elements will be minimal, avoiding errors due to shadows. Similar conditions are achieved on days with high clouds, where soft shadows are generated and do not significantly harm the captured information. It is recommended that the flight be conducted as long as the weather conditions allow; otherwise, you must interrupt the process, and wait for the right conditions or cancel the flight session and reschedule. 

To achieve reconstructions where PI geopositioning is required with a high level of accuracy, control points can be incorporated into the measurement, referring to the demarcation of milestones in the infrastructure, for which it is necessary to measure their GPS position with a specialized instrument. These points will then be incorporated into the photogrammetric process.

### 4.3. Data Processing

With the set of obtained images, we proceed to the processing of images. Using the structure from motion (SfM) method, it is possible to perform a photogrammetric reconstruction of the PI, obtaining digital three-dimensional models from the superimposition of images. SfM is based on machine vision algorithms that automatically correlate the camera orientation and positioning coordinates between the captured photographs, identifying points of interest and characteristic coincidences between the photographs. At this stage, if the control points are used, the control points must be introduced to improve the quality of the reconstruction and obtain high-precision geopositioning. The point cloud generated from SfM is densified using MultiView Stereo (MVS), which generates a dense, higher-resolution point cloud.

### 4.4. Reconstruction BIM Model

It is possible to perform the geometric reconstruction of a BIM model of the PI. The point cloud generated in the previous stage, together with the background of the infrastructure under study (CAD drawings, previous models, various antecedents of its structuring and materiality, etc.), will serve as the basis for the geometric reconstruction of the 3D model on BIM platforms. Independent of the chosen BIM software, it is important to generate a parametric model, with types of elements corresponding to the nature of the actual infrastructure and following the general guidelines of the existing documentation. The relevant modelling ordered and coded all the elements of the model, therefore a set of these elements must be ordered and detailed for the inventory.

### 4.5. Definition of Maintenance Actions and Automated Worksheet Creation

Based on the generated BIM model, it is possible to obtain and visualize the inventory of all the elements that compose the PI, according to their typology, materiality, and/or other variables of interest. To obtain and manage the data associated with the inventory in an orderly manner, it is necessary to order this information in spreadsheets (type MS Excel), defining and indicating the status criteria and maintenance actions for each type of PI element. For this type of evaluation, it is necessary to define standardized categories to facilitate the processes of evaluation and prioritization of maintenance actions. Numerical scales are recommended; for example, categorizations of values 1 to 5, where values 1 and 2 are assigned to those elements that do not require conservation, 3 and 4 to those that require conservation to be programmed, and 5 for those that need urgent conservation. The goal is to achieve the most automated form possible.

### 4.6. Definition of Maintenance Actions and Automated Worksheet Creation

**Figure 4:**
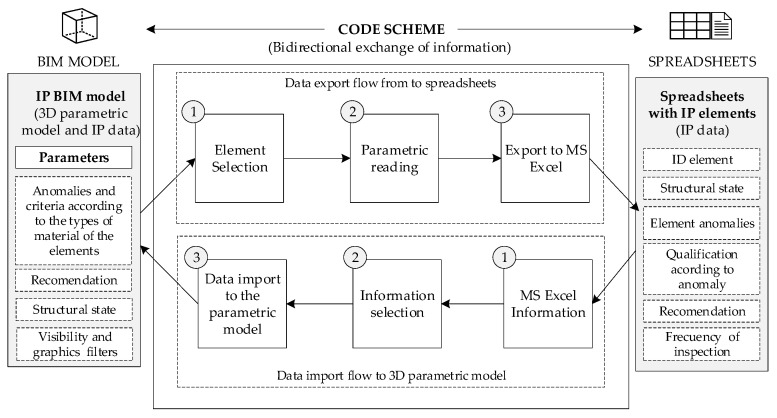
Proposed code scheme.

The stated criteria according to the typology of the elements must be incorporated as parameters to the BIM model. It will then be necessary to adapt or create parameters for the specific elements, along with actions for the graphical display to be obtained on the BIM platform (associated, for example, with display in colour scale according to a category, actions, and/or maintenance priorities, as required). To finish the process, the parametric BIM model is linked to the automated worksheet to obtain automatic bidirectional data updates. The use of visual programming tools allows for the automatic interconnection of BIM platforms with datasheets, using algorithms based on bidirectional workflows, thus obtaining an asset management tool for port infrastructures, particularly penetration springs. A code that allows for the bidirectional exchange of information between spreadsheets and the BIM environment was developed. A simplified schematic of the code created is shown in Figure 4.

## 5. Case of Application

**Figure 5:**
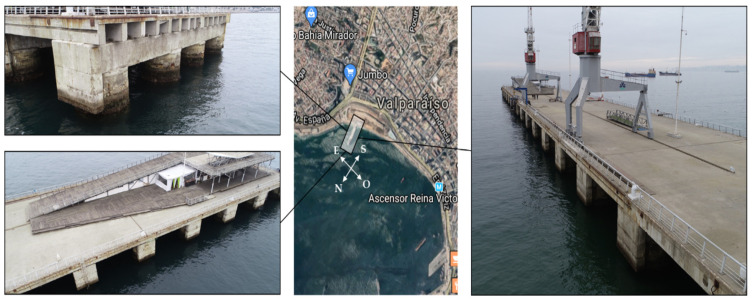
General description of port infrastructure case study (photographs by the authors. Central picture from Google Maps).

The proposed methodology has been implemented in port infrastructure, corresponding to a penetration dock built in 1912. The reinforced concrete structure was repaired in 1990, and no major repair work has been carried out since then. This dock is located in a seismic geographical area with the potential occurrence of tsunamis, and it is also exposed to tidal waves and strong waves, which, in winter season, force its closure. Figure 5 shows the location of the dock and the elements that make it up.

At present, the infrastructure is not in operation and is intended for recreational and sporting activities, therefore, there is a need to ensure its functionality for such activities to be carried out safely, as no maintenance is currently being performed on it.

**Figure 6:**
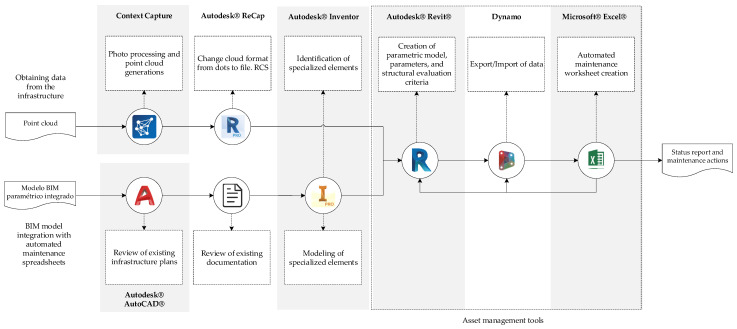
Flow for the generation of asset management tool.

It is important to maintain infrastructure in a highly aggressive environment where materiality is affected by the different pathologies described above. To meet this need, the implementation of a maintenance management tool is proposed based on an integrated BIM model with a maintenance proposal, facilitating the visualization of the elements and their structural state, categorized according to the type of items and repair activities required. Figure 6 details the tools and software used to meet the objective of this application, following the proposed workflow.

### 5.1. Photogrammetric Reconstruction and Development of the BIM Model

The IP is protected by a naval military zone, therefore, according to the regulations established at the national level, it must have a special authorization from the institution to which it belongs. In this case, the flight authorization was requested to the Port Captaincy of Valparaiso. 

A UAV DJI Phantom 4 Pro was used to take photographs, the characteristics of which are shown in Table 1.

Photographs are taken covering the entire surface and perimeter area of the pier with oblique and vertical photogrammetry, as described in the proposed methodology, obtaining a total of 148 photographs with an overlap of 80%. The registration was made at noon (between 12:00 h and 12:45 h) on a day with high cloudiness (ideal conditions to avoid shade in the structure). The UAV pilot toured the structure, maintaining the visibility of the UAV, to avoid risks associated with the management of the equipment very close to the seawater surface. The team did not record photographs under the infrastructure because, after reviewing the background, it was identified that the elements undercover are repetitive elements of the same geometry as the exteriors (as captured by the UAV), therefore the photographs taken would be sufficient for the generation of the 3D model. Table 2 presents the setting parameters.

**Figure 7:**
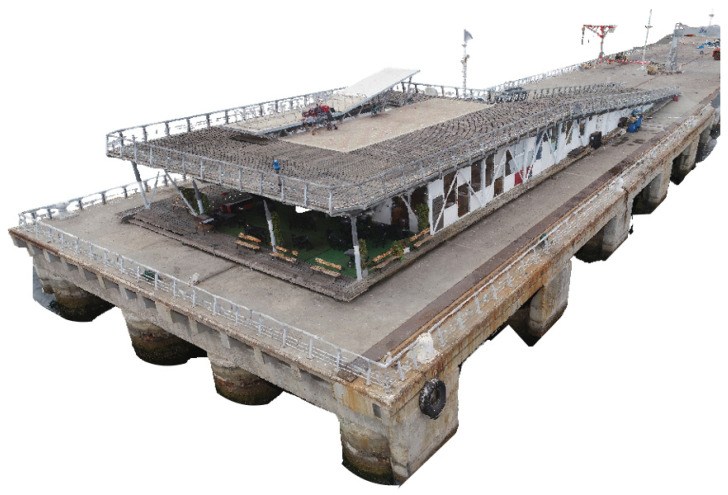
Point Cloud Context Capture.

For the application of photogrammetry and 3D scene reconstruction algorithms (SfM-VSM algorithms), ContextCapture (Bentley) software was used, allowing for generation of a digital 3D model from a set of imported photographs, the detection of camera parameters, and the pose of each image entered. Through aerotriangulation, the program detects key points and identifies similar points in different images. With this information, it is possible to triangulate the positions of each of the key points using the pose delivered by the UAV sensors, obtaining a cloud of scattered points with the location and corrected orientation of each photograph obtained by the UAV. Figure 7 presents part of the structure reconstructed using ContextCapture software.

BIM structural modelling software is required for the reconstruction of the 3D model. In this application case, we used Revit^®^ (Autodesk^®^, Mill Valley, CA, USA), which stands out for the modelling of elements from custom families and user-defined parameters, allowing for a precise reconstruction, which, added to its massive use, interoperability, and data export properties, make it an attractive asset management software [37]. Before 3D modelling, Autodesk^®^ ReCap was used to convert the point cloud format to a file RCS-compatible with Autodesk^®^ Revit. Infrastructure documentation (general CAD drawings, reports, and sheets, with outdated information) was reviewed to identify parameters and technical considerations. The elements of the infrastructure were modelled on Autodesk^®^ Revit based on the point cloud (RCS) and imported CAD drawings.

**Figure 8:**
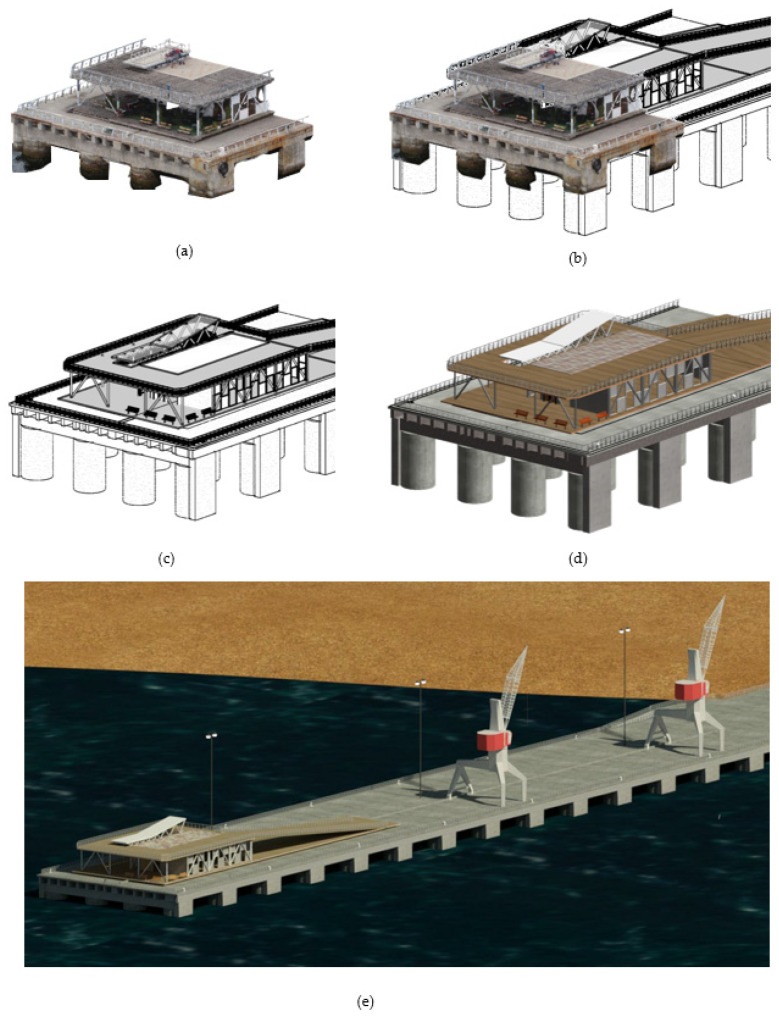
Reconstruction of the parametric IP BIM model in Autodesk. Revit, based on cloud of points and CAD planes. (**a**) IP Point cloud; (**b**) Initial reconstruction of IP BIM model based on point cloud; (**c**) Development of IP BIM model; (**d**) Reconstructed IP BIM model; and (**e**) Overview of reconstructed IP BIM model in environment.

The process of modelling based on the cloud of points and CAD planes starts with the creation of axes and reference planes, which will serve as a guide to locate the elements. Customized families were created for the creation of reinforced concrete elements (slabs, beams, piles, slab protection), railings, and crinoline staircases. To create a generic model for the berths (elements for boat moorings), we used the software Inventor^®^ (Autodesk^®^, Mill Valley, CA, USA), which allows us to create custom models exportable to Autodesk^®^ Revit with more complex geometries (since Revit has a traditional construction element approach). Figure 8 shows the reconstruction of the 3D model, from the import of the point cloud and CAD drawings to the final model.

**Figure 9:**
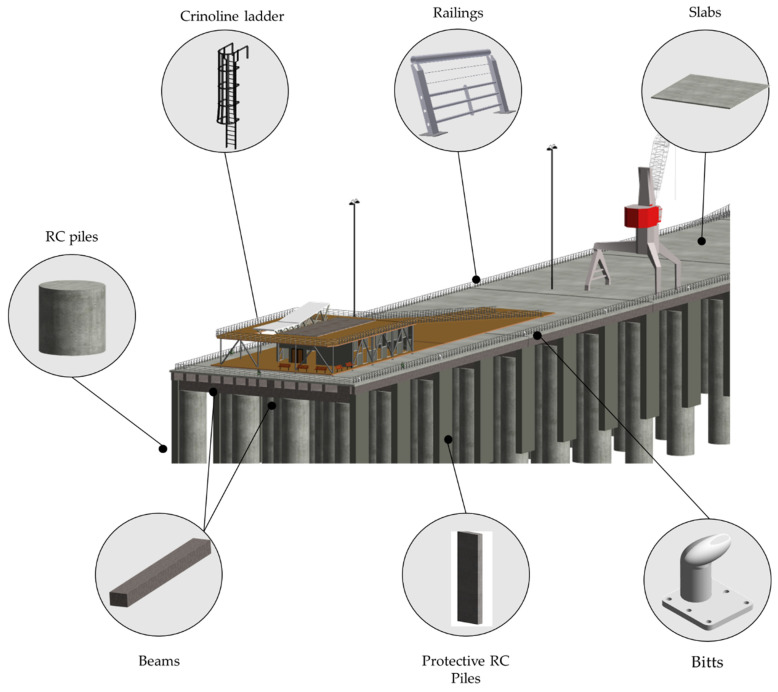
Elements of port infrastructure.

Figure 9 shows the generated BIM 3D model and its main elements: longitudinal beams and crossbeams, slabs, batteries and protection batteries, metal elements such as railings, crinoline staircases, and specialized elements such as bitts.

### 5.2. Definition of Anomalies and Maintenance Activities According to the Type of Elements

To carry out a structural evaluation of the elements that make up the infrastructure, evaluation and categorization criteria were defined, together with maintenance activities. The Design, Construction, Operation and Maintenance Guide of the Port Works Directorate of Chile defines the main anomalies according to the types of material of the elements. Table 3 and Table 4 present the anomalies and degrees of deterioration of the main materials, reinforced concrete, and metal elements.

Each degree of deterioration is evaluated with a rating ranging from 1 to 5 for each anomaly, which was assigned to visually facilitate the degree of deterioration according to the type of element (Table 5).

The criterion for determining whether the element requires maintenance will be conditioned by the maximum note obtained between anomalies, where 1 and 2 do not require, 3 requires but can wait, and 4 and 5 require urgent intervention considering that the element is bad or very bad (Table 6).

In addition, maintenance activities are defined for the elements in question. For reinforced concrete elements, there is the repair of cracks (surface or deep fissure sealing), steel replacement-concrete, structural repair, cleaning joint expansion in slabs, cleaning, and painting for railings and bites.

### 5.3. Creation of an Asset Management Tool

**Figure 10:**
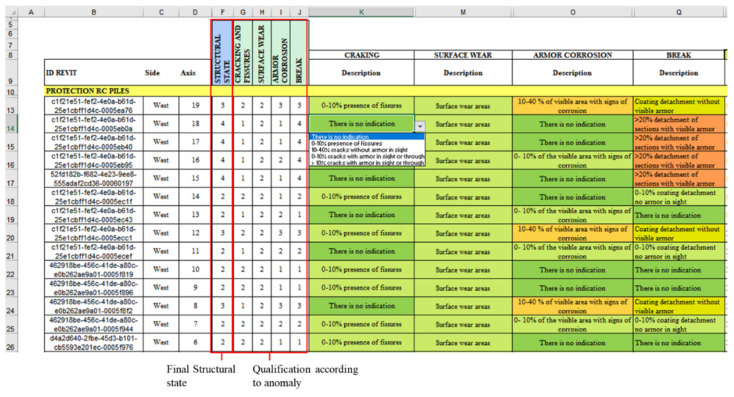
Automated MS Excel^®^ form.

The anomalies presented, the evaluation criteria, and the maintenance activities described were linked and automated in an MS Excel^®^ spreadsheet; therefore, when modifying the criteria of the anomalies, the structural condition of the element was automatically updated, determining the urgency of maintenance. Figure 10 shows an extract from the automated worksheet, where, from the created drop-down lists, it is possible to modify the state of the item concerning an anomaly, its anomaly rating, and, in turn, the structural state of the element corresponding to the maximum note of each of them.

The infrastructure asset management tool is based on the integration of the parametric BIM model with the automated spreadsheets in MS Excel^®^, so that each element represented in the model contains updated information regarding its anomalies, structural condition, and recommended remedial activities. To achieve this goal, a reliable information flow between Autodesk^®^ Revit^®^ and MS Excel^®^ is required. For this information flow, Dynamo was used, an extension of Autodesk^®^ Revit^®^ based on visual programming, which, from visual elements knows as “nodes”, allows for the creation of custom algorithms to process data or create complex geometries.

**Figure 11:**
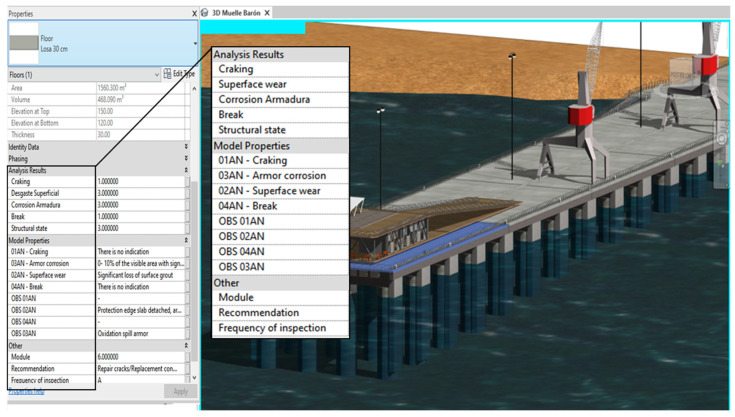
Parameters created for reinforced concrete elements.

Before this step, it was necessary to develop a BIM model in Autodesk^®^ Revit and create parameters that would allow for storing the information of the automated worksheets. Therefore, parameters associated with the anomalies of the elements were created according to their material and degree of deterioration. Figure 11 shows, as an example, the parameters created for the infrastructure slabs, elements on which the explanation of data import/export flows will be based.

**Figure 12:**
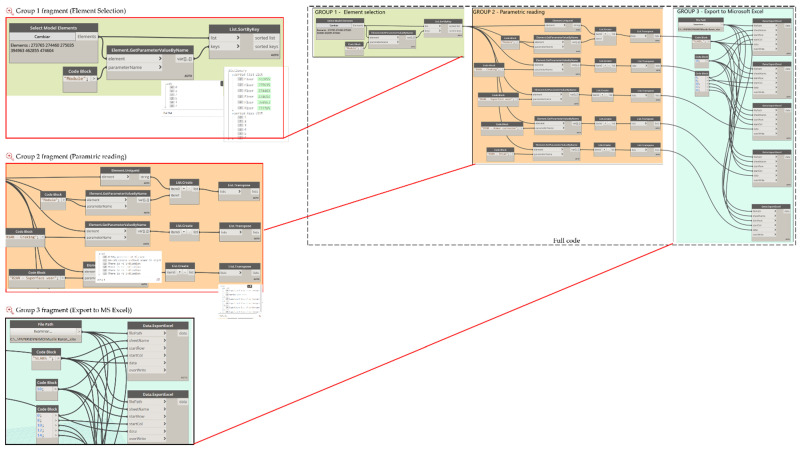
Data export flow from Autodesk^®^ Revit^®^ and MS to Excel–Slabs.

To facilitate the visualization of the elements in the automated spreadsheets, it is required that the information be entered in an organized way, without altering its previous configurations, therefore additional parameters associated with the location of the elements were created. For example, concrete slabs 1 to 6 have been listed using the parameter created called *Module*. Figure 12 shows the export flow of these six elements, which has been organized into three groups. Group 1 selects and organizes the model information to be exported. Dynamo has a series of nodes that allow selecting the elements, by family and by type, among others. In this case, since there are only six elements, the *Select Models Elements* node was used to select the elements directly in the model. The *SortByKey* node allows you to sort lists according to a specific parameter, for which use can be made of the created location parameters. Therefore, this node has as its input the list of elements to classify and the parameter for their classification; the parameter is specified using the *GetParameterValueByName*, which obtains the information from the specified parameter of the elements; however, these are disordered. In this way, the *SortByKey* code allows the output of group 1 to be two lists ordered according to the indicated parameter, and list 0 contains the six elements of the slab ordered correlatively according to its “Module”, a parameter contained in list 1.

Group 2 reads the parameter information you want to export to the automated spreadsheets. Therefore, from the output of group 1, the list of ordered items is used (list 0), which is connected as an input to the *GetParameterValueByName* nodes specifying the different parameters of the elements and, additionally, reads the *UniqueId,* which obtains a list with the unique ID of the elements (which is generated by the default software). The information obtained from each of the parameters is stored in lists through the *ListCreate*, node; since this node returns the information in lists organized in rows, it is necessary to use the *Transpose* node to transfer the data, so that, when exporting parametric data to spreadsheets, each parameter is organized by columns.

**Figure 13:**
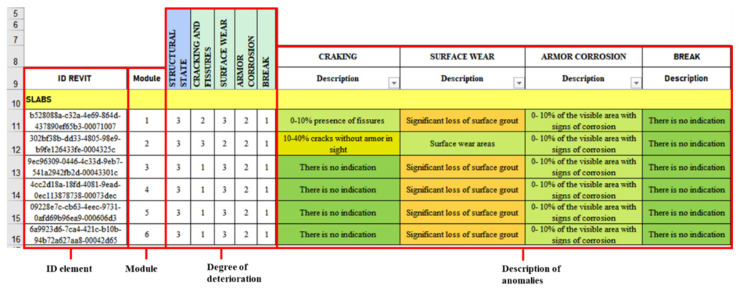
Automated inspection form—Elements: Slabs.

In group 3, the *Data*, the *ExportExcel* node is used for data export; this node indicates the path of the target file (FilePath node), the sheet on which the data will be written (SheetName node), and the row and column from which the data will start to be filled (StartRow and StarCol nodes, respectively). The numbering of rows and columns starts from (0,0), that is, row 0 corresponds to row 1 of MS Excel, column 0 to column A, etc. The described flow allows for sending all the information of the parameters associated with the location and anomalies of the elements to the automated worksheets in an organized manner, which is also modifiable from the drop-down lists created, as shown in Figure 13. 

**Figure 14:**
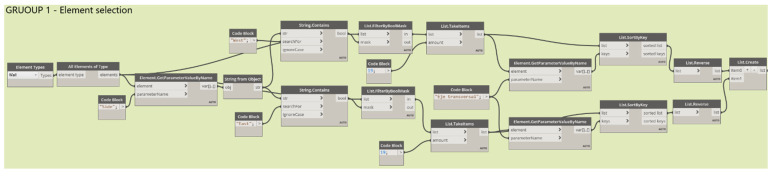
Data export flow from Autodesk^®^ Revit^®^ and MS to Excel–Protection RC Piles Group 1.

Similarly, the flow was performed for the other elements, where in group 1 the information is selected, in group 2 the UniqueID is specified, as well as the parameters of location and anomalies of the elements, and, in group 3, the export of the elements themselves is carried out. Figure 14 shows an example where the flow of group 1 is more extensive. This case corresponds to the battery protections (modelled as a wall), where the number of elements is greater, so other nodes are used to sort the information according to how you want to visualize it in the forms. Prior to this, two parameters were created that will allow organizing the elements in the forms: one corresponds to the “Side”, which can be East or West, and the other corresponds to the “Transversal axis”, which goes from A–1 to A–19.

**Figure 15:**
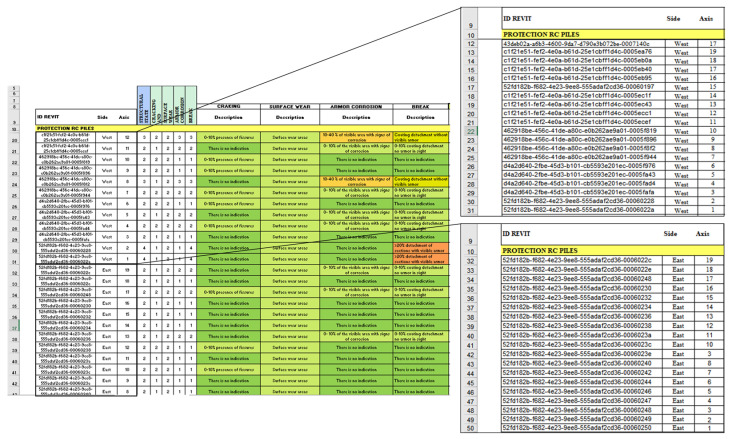
Automated inspection form—Elements: Protection RC Piles.

In this case, the selection of elements is made according to their typology through the node *Categories* and *AllElementOfType*; in this way, all the walls created in the model are selected. To filter only those that correspond to the battery protections, the node is obtained. Parameter *ByName* was used to indicate the parameter “Side”, followed by a sequence of nodes that reaches *FilterByBoolMask,* which gives a list of Boolean (True/False) values that allows for the generation of two separate lists of those elements on the east and west sides. However, these lists are also ordered correlatively according to the transverse axis; therefore, the nodes *GetparameterValueByName* and *ListSortByKey* are used again, now obtaining two separate lists that are exported to the automated forms, where the elements are grouped according to the “Side” and sorted according to the “Transverse Axis”, as shown in Figure 15.

In addition, to generate a bidirectional flow between MS Excel^®^ to Autodesk^®^ Revit^®^, Dynamo algorithms were created that allow the import of data from MS Excel^®^ to Autodesk^®^ Revit^®^. Therefore, the information that is modified in the automated spreadsheets will be uploaded to the BIM model, keeping the model updated according to inspections carried out. Similar to export flows, these flows have been grouped into three parts.

**Figure 16:**
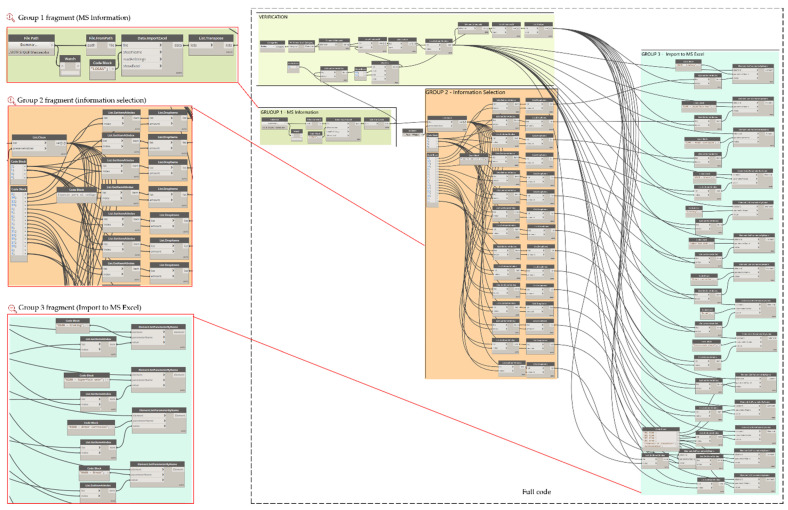
Data import MS Excel^®^ to Autodesk^®^ Revit^®^.

Figure 16 presents an example of the scheduled flow for importing the slab inspection data. In group 1, the data in the MS Excel^®^ spreadsheet are read through the *Data ImportExcel* node, in which the path of the file and the name of the sheet containing the data to be imported must be specified. The output of this node returns the information in a series of lists by rows, therefore the *List transpose* node is very useful to organize the information as it comes from the original worksheet. In group 2, the information to be read from the automated spreadsheets is specified and separated, and the *GetItemAtIndex* node allows us to indicate the index of the list containing the parameter information, which allows us to generate separate lists of the parametric information, which will be loaded to the parameters specified in group 3 via the *ParameterByName.*

**Figure 17:**
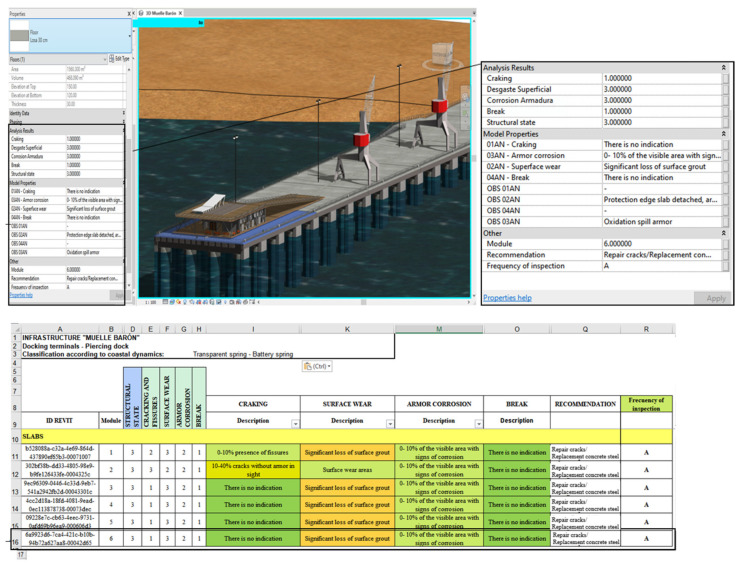
Example display data in parametric BIM model.

In this way, when selecting an element in the model, it is possible to see, in the properties tab, the updated inspection information, its structural condition, the frequency of inspection, and the recommended activities according to the material of the elements, information matching the automated spreadsheets (Figure 17).

**Figure 18:**
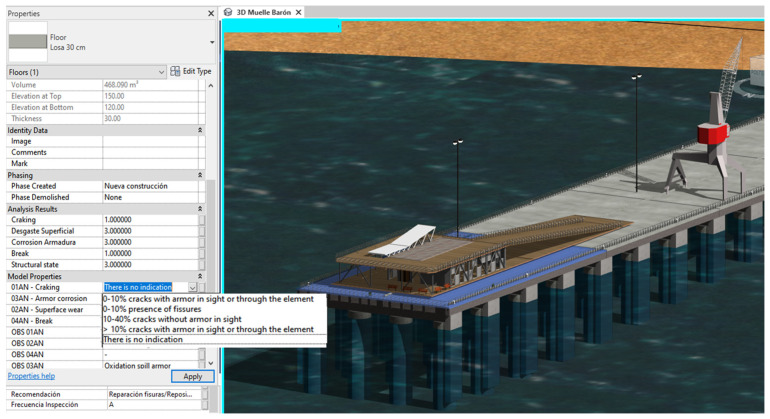
Criteria for anomalies in Autodesk^®^ Revit^®^.

For the asset management tool to be managed from the model and the MS Excel worksheet to be updated, copies of elements have been created to which parameter values created in Autodesk^®^ Revit^®^ have been assigned, to display in the parameters box a list of all possible states of each anomaly as shown in Figure 18; the BIM model and the automated spreadsheets are modifiable, allowing a two-way flow of information from Autodesk^®^ Revit^®^ to MS and Excel.

### 5.4. Display of Final BIM Model

**Figure 19:**
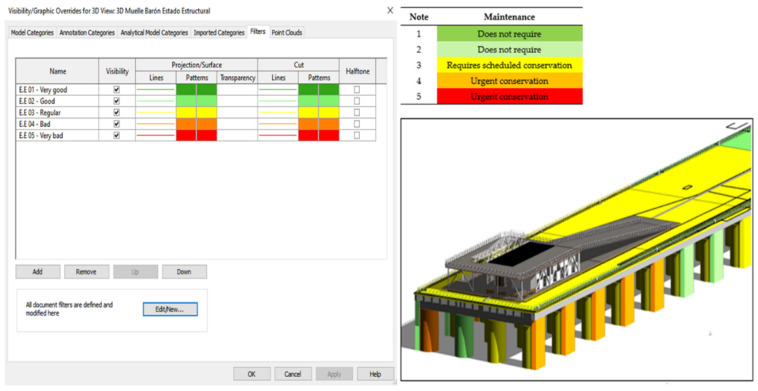
Filter Visibility/Graphics created in Autodesk^®^ Revit^®^.

To facilitate the visualization of the structural state of the elements of the infrastructure, graphic visibility filters were created in Autodesk^®^ Revit^®^ according to the structural state of the element to determine the need and urgency of maintenance of each element. In this way, it is possible to visualize the infrastructure with the corresponding colours, which vary according to the information record after each inspection (Figure 19). 

**Figure 20:**
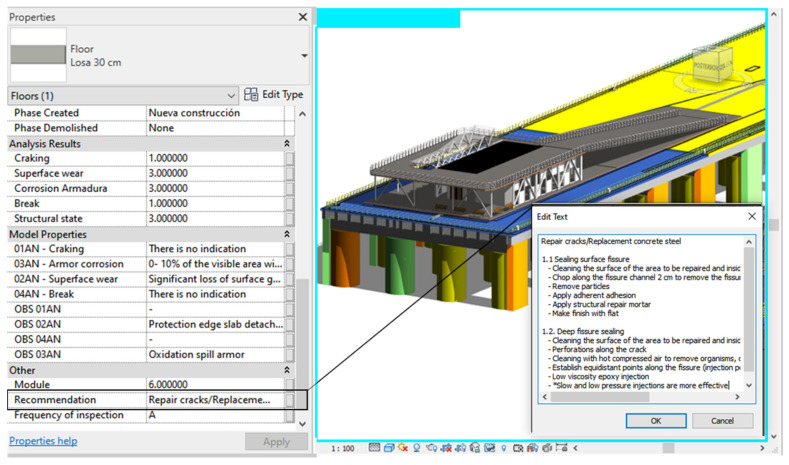
Inspection frequency and maintenance activities—Element: Slab.

Additionally, two parameters have been created for all elements of the Autodesk^®^ Revit^®^, the infrastructure, one of which indicates the frequency of inspection of the element and, another, the recommended maintenance activities that must be validated and/or supplemented by the engineer in charge of the inspection. Therefore, the performance of the recommended maintenance activities will be conditioned by the rating of the particular item. Figure 20 shows an example of a slab cloth in which, in addition to the structural state properties, it is possible to visualize that an annual inspection, represented with the letter A, should be carried out, as well as the recommended maintenance activities. Since this element has a regular structural state, the indicated maintenance activities do not require more urgency so that they can be programmed.

In this way, it is possible to visualize, in the model, both the structural state of the elements, their rating, the location parameters, the inspection frequency, and the recommended activities aligned to the automated forms that facilitate the inspection of the port infrastructure, keeping both platforms constantly updated.

## 6. Discussion

In the literature, works related to the method proposed in this research, where the use of UAVs (as well as in combination with scanners), point cloud, and BIM for facility management is integrated. The applications are focused on knowing, at different levels of detail, the condition of structures such as bridges [44] and towers [45], as well as uses for heritage BIM (HBIM) [46,47]. The capture of damage in these works is obtained from non-invasive methods in contact with the structure and the identification from images obtained with the UAV. Thus, these articles offer different alternatives for capturing damage and parameters of interest to input into a BIM model. However, no detail of the general process applied would allow for replication of the process in other situations. Moreover, no applications to maritime works were found.

This research, along with being applied in a case not seen before in the literature, offers a generic and replicable method for other structures. In addition, our proposal seeks to be a management tool, allowing its exploitation both for users who do not use BIM (using only the structure status registration form) and for BIM users on the parameterized model. Thus, this is intended to be a methodological work focused on applied research. It differs from other related papers, generating a generic and replicable methodology for other structures. Also, we illustrate the application of the method with a detailed applied case, facilitating understanding and replication for researchers and practitioners.

The proposed recommendations provided an orderly acquisition of photographics through UAVs for the generation of point clouds. With this, together with the technical background and drawings of the project, it was possible to reconstruct a parametric BIM model of the PI. Although a BIM model of all elements of the PI was performed, and the current conditions of the PI with respect to the outdated plans and technical background were rectified, limitations in the model’s photogrammetric reconstruction were identified.

Despite the high resolution of the images acquired, the structure’s sea movement and shadows did not allow for the correct reconstruction of all the exterior pillars. In addition, it was not possible to access or reconstruct the bottom side of the infrastructure due to the swell and the absence of light. Taking photographs in these conditions with UAVs represents a risk for the equipment. In addition, a reconstruction with pictures is not possible in these conditions (low light and many shadows). Other reconstruction techniques of existing infrastructure could be used (such as Lidar sensors or 3D scanning). However, the team did not have this equipment. In addition, the research team aimed to use low-cost tools to facilitate the use of the methodology; therefore, these other tools were discarded.

An automated Excel spreadsheet was developed, containing information on all the elements of the PI, their ID coding, structural condition, and maintenance measures. In addition, the BIM model was able to incorporate all this information, together with the visualization of the color scales associated with the structural states. The code created allows for the bidirectional connection of data between both work environments. Thus, the maintenance manager will perform his inspections from Excel or Revit, and the information will be updated and synchronized in the other platform. Moreover, if new elements are created in excel or Revit, the other platform will recognize them and update the information, maintaining the bidirectional synchronization.

The application of our methodology and the case study involve certain obstacles. The main difficulty lies in the complete visualization of the structure, where it is not possible in certain cases. In the case study, it was not possible to access the pier’s piles, where, in addition, there were not good lighting conditions. This problem could be solved by accessing old plans of the structure, which are not always available. Another obstacle is the reconstruction of complex elements to model with photogrammetry. In this case, the cranes of the pier are elements that, to be well represented in the model, require a special flight strategy. To make the application of the methodology efficient, it is important to be clear about the detailed requirement of the elements to be controlled. In cases where a detail of the component is not required, a geometrically simplified version can be chosen, as in the case of the dock cranes.

## 7. Conclusions

This investigation identified the main deficiencies and difficulties of the plans for the maintenance of port infrastructures that are associated with an inefficient inventory of elements, with the lack of existing information, erroneous digitization in the transfer of information, little use of asset management tools, and difficulty of access. Thus, processes involving a maintenance plan, such as inventory generation or infrastructure inspections, are time-consuming and unreliable. This information helped to clarify the need to maintain this type of infrastructure to prevent progressive damage over time that would lead to higher repair costs.

An asset management tool was developed using technologies and methodologies as potential tools to optimize traditional processes involving maintenance plans. A code was developed and implemented for bi-directional exchange between the BIM environment and the traditional IP management spreadsheets. From this point, the advantages of the proposed methodology are clearly and objectively shown, managing to efficiently capture the information needed to generate clear, orderly, and dynamic asset management.

The proposed methodology was implemented in port infrastructure, particularly a penetration dock where a large number of the elements that make up the dock are immersed in seawater and are affected by salinity and the marine environment, developing various pathologies which must be controlled to avoid progressive deterioration of the infrastructure. The structural state of the elements was obtained from visual inspection, and the categorization of structural damage along with these maintenance activities was defined according to material, information that can be easily accessed in the digital model and in the MS Excel spreadsheets. Through visual programming, it was possible to generate a two-way flow of automatic import and export of information, which allows for the digital model to be kept up to date and with a clear visualization of the structural state of each element and its maintenance activities, presenting the possibility of handling this tool from the MS Excel spreadsheets or from the same model generated in Autodesk Revit. The tool developed proves to be very useful for optimizing maintenance activities and keeping an up-to-date record of the activities carried out. To determine the structural condition of the infrastructure in greater detail, it is advisable to supplement the results obtained in visual inspection with laboratory or in situ tests, whether destructive or nondestructive. Among the applicable tests are the compressive strength test, the strength of the reinforcements, and carbonation, among others. In this way, it will be possible to know the current state of the elements and define maintenance actions that complement those already established, generating a complete maintenance plan that addresses the current deficiencies of the elements of the infrastructure.

The digital reconstruction of a historic infrastructure by photogrammetry with UAVs allows for the centralization of documentation and dispersed existing information, which, added to the interoperability properties and an automation tool, allows for the development of a management tool for the maintenance of the infrastructure, which concentrates the characteristics and dimensions of the elements, their current structural state using a color scale according to the structural evaluation, the urgency of intervention, and the recommended maintenance activities required according to the anomalies and deterioration of each element, contributing significantly to FM activities.

As a future line of research, we propose to study the efficiency of the developed asset management tool and its usability over time in real applications to optimize traditional maintenance methods. In this sense, the new methodologies and technologies that accelerate the digital transformation in construction would allow, in the following years, the planning of predictive maintenance for infrastructures. The use of digital twins or digital twins in industry 4.0 is based on the identical virtual representation of a real environment in all its aspects, in which it is possible to simulate scenarios of all kinds and to know the behaviour that the infrastructures would have without affecting the behaviour, which would make it possible to define maintenance activities avoiding the development of progressive damage, speeding up current processes, increasing resource efficiency, and generally facilitating decision-making associated with different aspects, opening up possibilities for the development and implementation of predictive maintenance of infrastructures by drawing on the full potential of emerging technologies and methodologies in the AECO industry.

## Figures and Tables

**Table 1:** Technical characteristics UAV DJI Phantom 4 Pro.

Resolution [MP]	Opening Range	Focal Length [mm]	Sensor [mm]
20 (5672 × 3648)	f/2.8–11	8.8	12.83 × 7.22-CMOS

**Table 2:** Setting model parameters.

Parameter	Value
Number of photos uploaded	148
Number of photos used	115
Percentage of photos used	78
Processing time	2 h 99 min
GSD	6.45 mm/px
Model scale	1:19
Image dimensións	5472 × 3078 px
Total Tie Points	28,242
Average Tie Points per image	1193
Average RMS error	0.47 px
Minimun RMS error	0.01 px
Maximum RMS error	1.77 px

**Table 3:** Anomalies and criteria elements of reinforced concrete.

Anomalies	State	Description
(I) Cracking	Very good	There is no indication
	Good	0–10% presence of fissures
	Regular	10–40% cracks without armour in sight
	Bad	0–10% cracks with armour in sight or through the element
	Very bad	>10% cracks with armour in sight or through the element
(II) Surface wear	Very good	There is no indication
	Good	Surface wear areas
	Regular	Significant loss of surface grout
	Very bad	>20% loss of visible coating/armour
(III) Armour corrosion	Very good	There is no indication
	Good	0–10% of the visible area with signs of corrosion
	Regular	10–40% of visible area with signs of corrosion
	Bad	>40% of visible area with signs of corrosion
	Very bad	Section loss
(IV) Break	Very good	There is no indication
	Good	0–10% coating detachment, no armour in sight
	Regular	Coating detachment, without visible armour
	Bad	>20% detachment of sections with visible armour
	Very bad	>10% element break or failure

**Table 4:** Anomalies and criteria metal elements.

Anomalies	State	Description
(I) Deformation	Very good	There is no indication
	Good	0–10% dents without damage to corrosion protection
	Regular	10–40% dents with damage to corrosion protection
	Bad	>20% loss of element symmetry axes
	Very bad	>10% section loss/element break
(II) Corrosion	Very Good	There is no indication
	Good	0–10% of visible area with signs of corrosion
	Regular	10–40% of the visible area with signs of corrosion
	Very bad	>40% of visible area with signs of corrosion
(III) Loss of corrosion protection	Very good	There are no indications
	Good	0–10% of the area visible without protection
	Regular	10–40% of the area visible without protection
	Bad	>40% of the area visible without protection

**Table 5:** Criteria note elements.

Criteria Note Elements	Rating
Very good	1
Good	2
Regular	3
Bad	4
Very bad	5

**Table 6:** The criteria for determining whether the item requires maintenance.

Maintenance	Rating
Does not require	1
Does not require	2
Requires scheduled conservation	3
Urgent conservation	4
Urgent conservation	5

## Data Availability

Not applicable.
